# Supplementary figures and images for: Structural and functional studies of the first tripartite protein complex at the Trypanosoma brucei flagellar pocket collar
Source: PLoS Pathog. 2021 Aug 2;17(8):e1009329. doi: 10.1371/journal.ppat.1009329 (PMC8360560; doi:10.1371/journal.ppat.1009329)

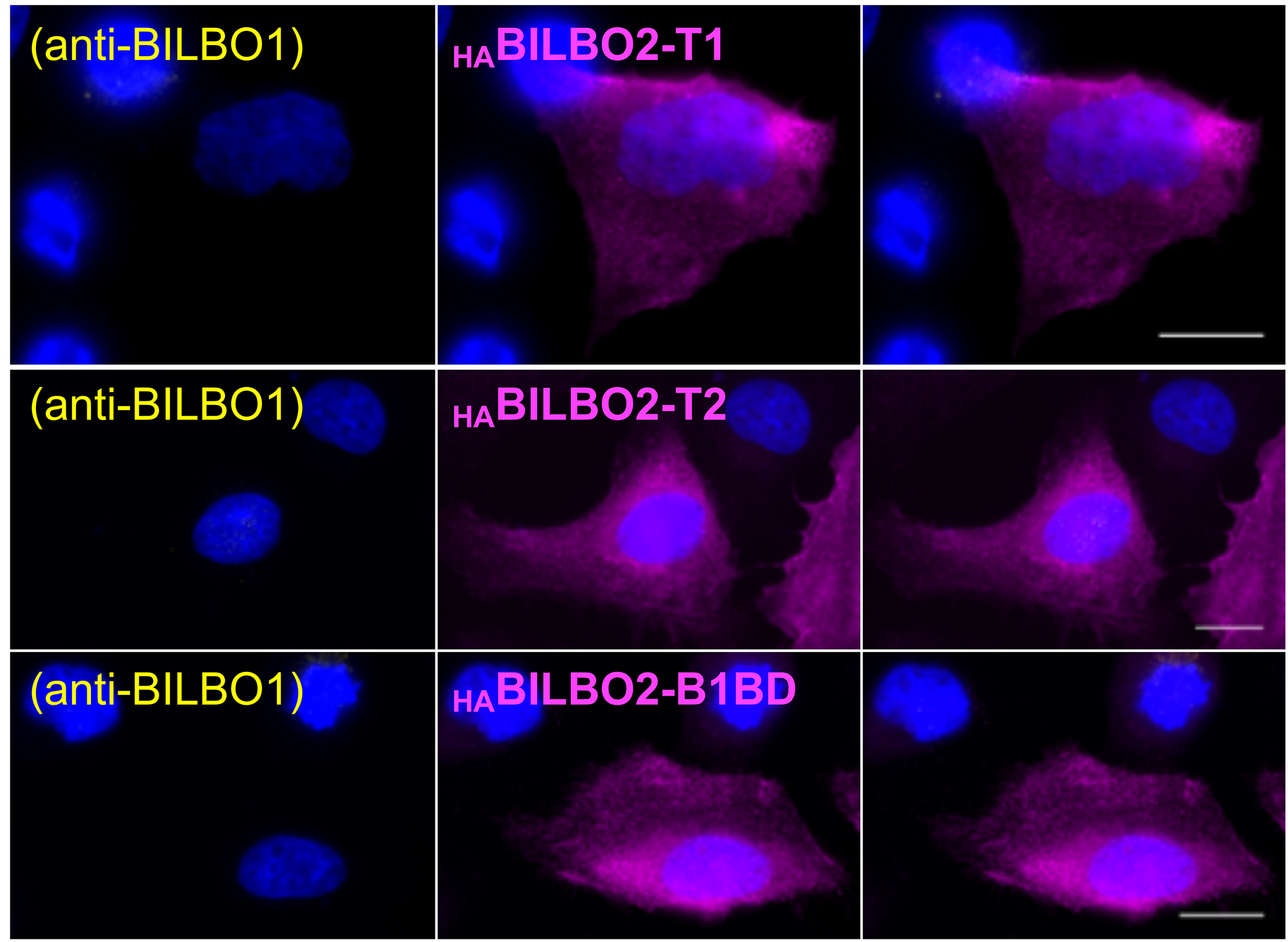

Supplement: S1 Fig — (TIF) [file ppat.1009329.s001.tif]

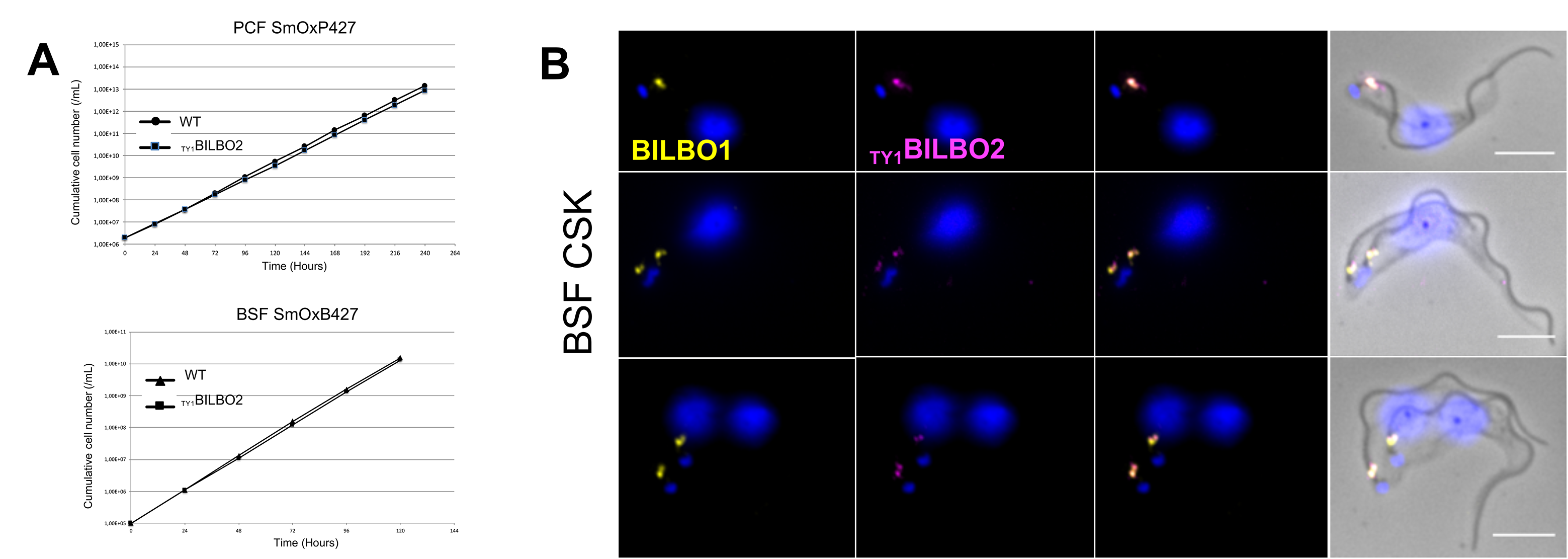

Supplement: S2 Fig — A. Growth curves of PCF and BSF expressing endogenous tagged TY1BILBO2. B. Co-immunolabelling of BILBO1 and TY1BILBO2 on detergent-extracted cells using anti-BILBO1 and anti-TY1 antibodies. Scale bars, 5 μm. (TIF) [file ppat.1009329.s002.tif]

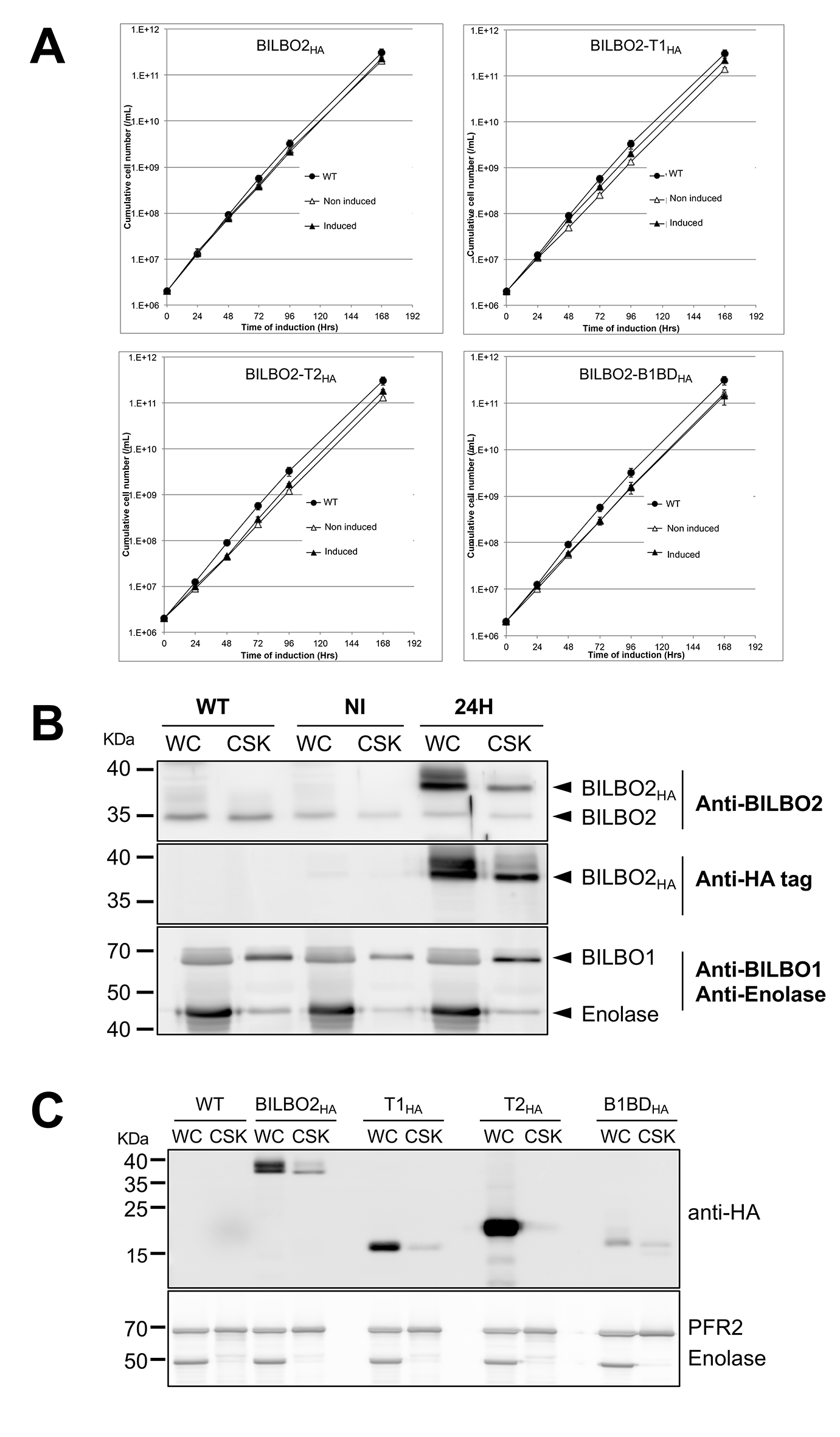

Supplement: S3 Fig — A. Growth curves of WT PCF cells, and non-induced and induced cells for ectopic expression of BILBO2HA, BILBO2-T1HA, BILBO2-T2HA, and BILBO2-B1BDHA. B. Western blot analysis of the expression level of BILBO2 and BILBO2HA. Whole cell (WC) or detergent-extracted cytoskeleton (CSK) were labelled using the anti-BILBO2 polyclonal antibody (that recognises WT BILBO2 and induced BILBO2HA) and the anti-HA tag antibody that recognises induced BILBO2HA only. Loading and detergent-extraction controls were anti-BILBO1 and anti-Enolase. C. Western blot analysis of the level of expression of BILBO2HA and domains in WC and CSK. Loading and detergent-extraction controls were anti-PFR2 and Enolase. In A, B and C, cells were induced for 24H with 1 μg.mL-1 tetracycline. (TIF) [file ppat.1009329.s003.tif]

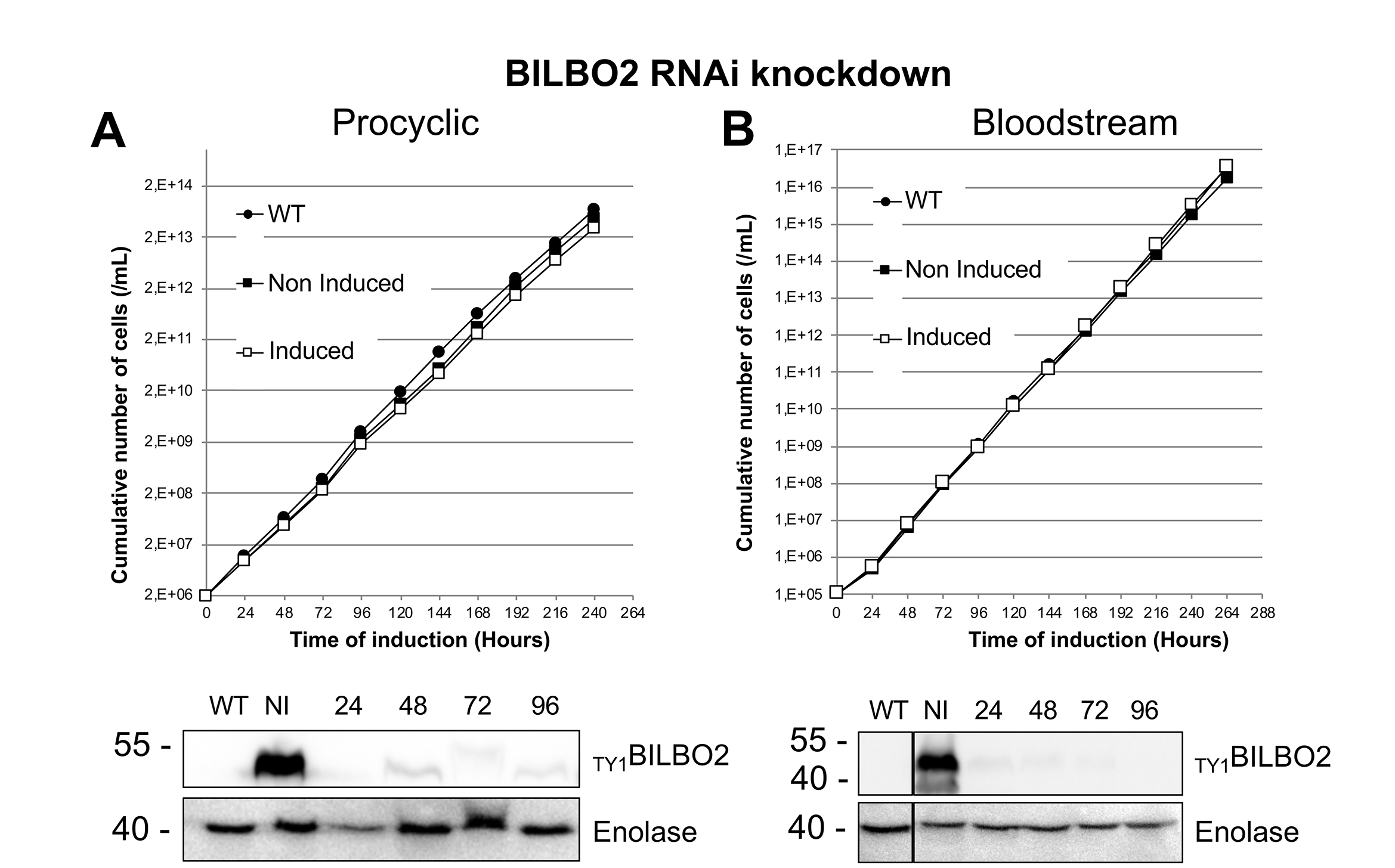

Supplement: S4 Fig — (TIF) [file ppat.1009329.s004.tif]

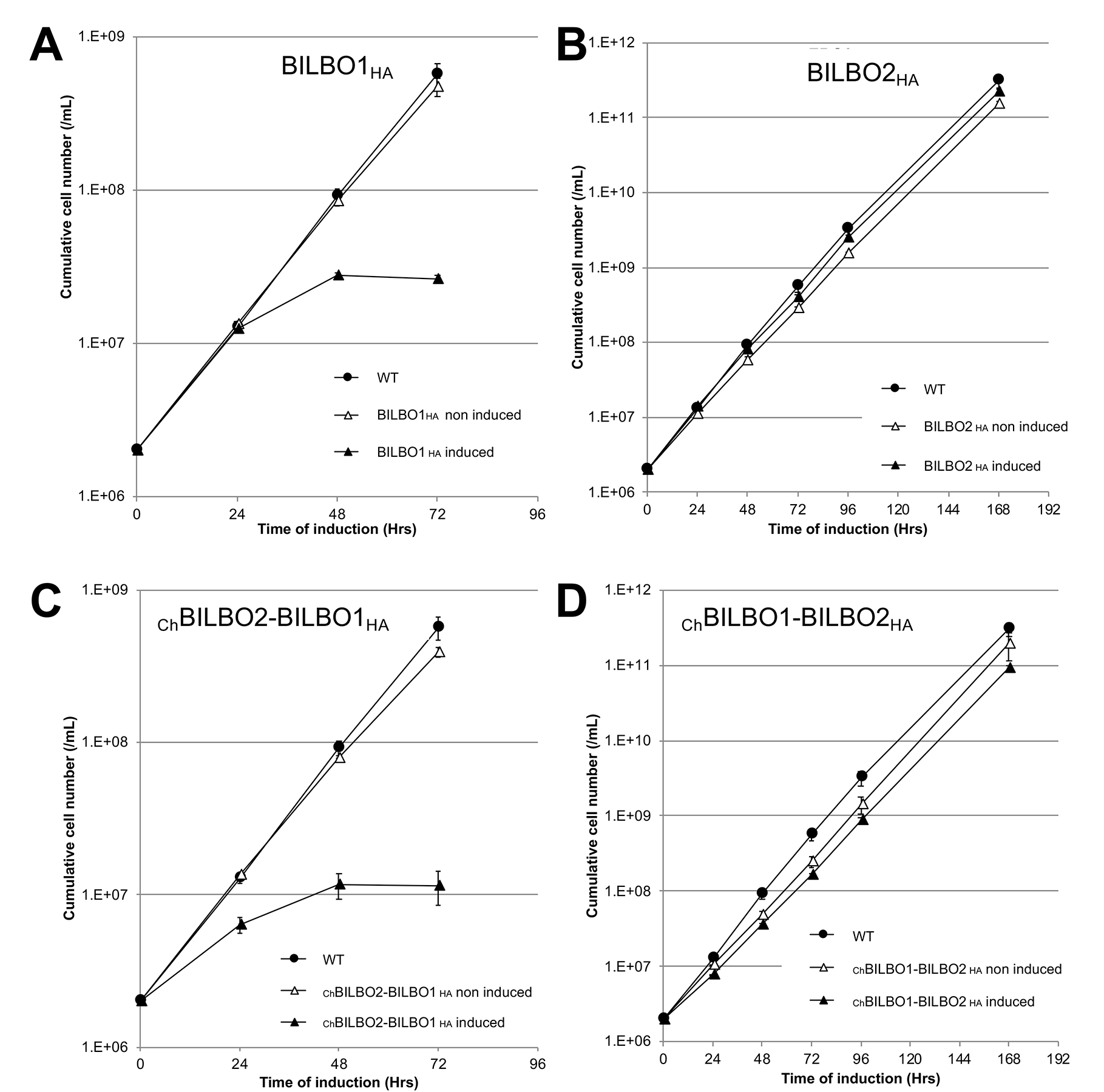

Supplement: S5 Fig — Growth curves of WT PCF cells, and non-induced and induced cells for ectopic expression of BILBO1HA (A) and BILBO2HA (B) and chimeric BILBO2-BILBO1HA (C) and BILBO1-BILBO2HA (D). (TIF) [file ppat.1009329.s005.tif]

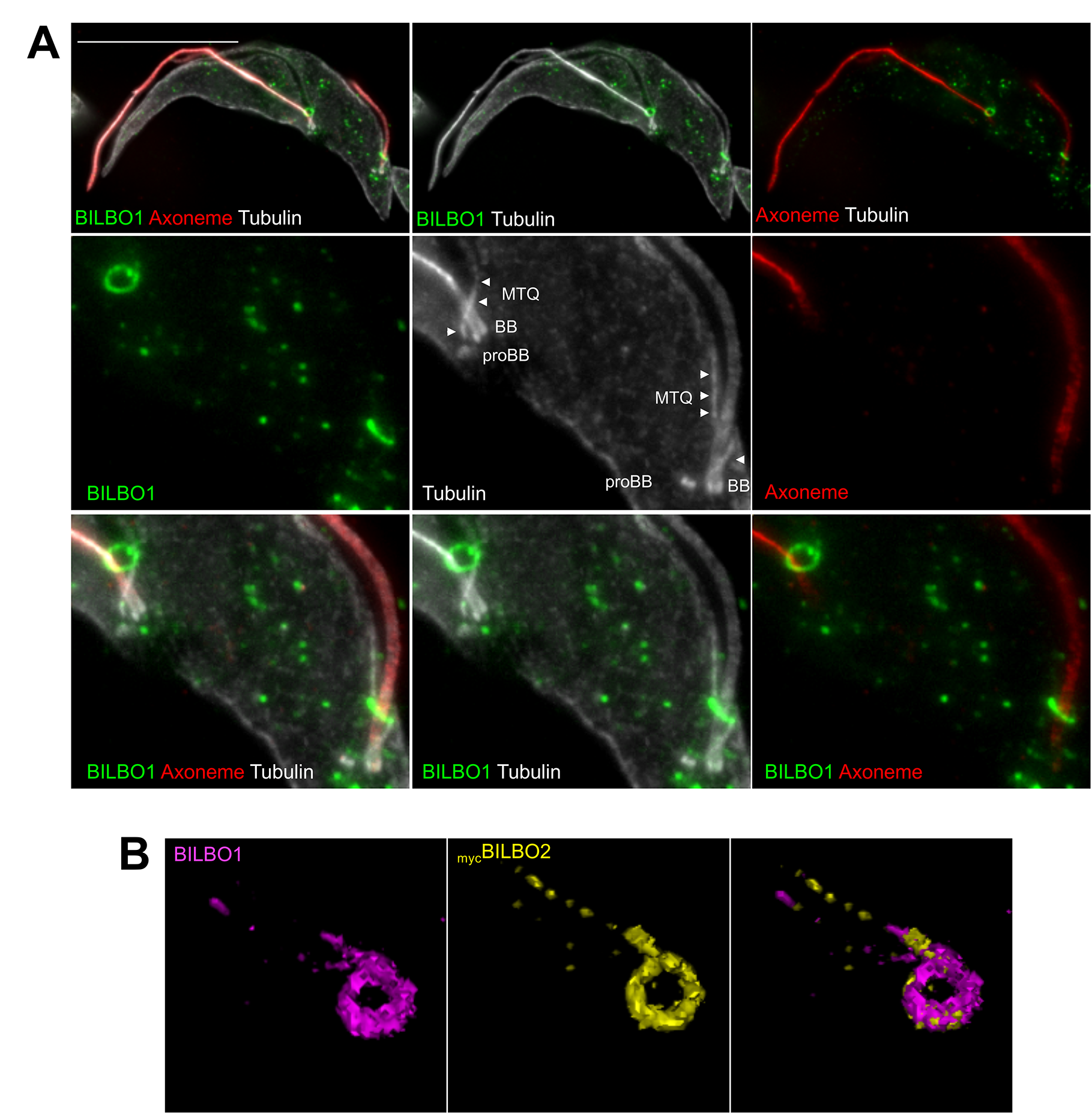

Supplement: S6 Fig — A. Epifluorescence image of U-ExM triple labelling of tubulin, BILBO1 and TbSAXO, an axonemal protein. The arrowheads indicate the MTQ. Scale bar, 20 μm. B. 3D rendering of confocal analysis of U-ExM co-labelling of BILBO1 and mycBILBO2 that were used to generate S1 Movie. (TIF) [file ppat.1009329.s006.tif]

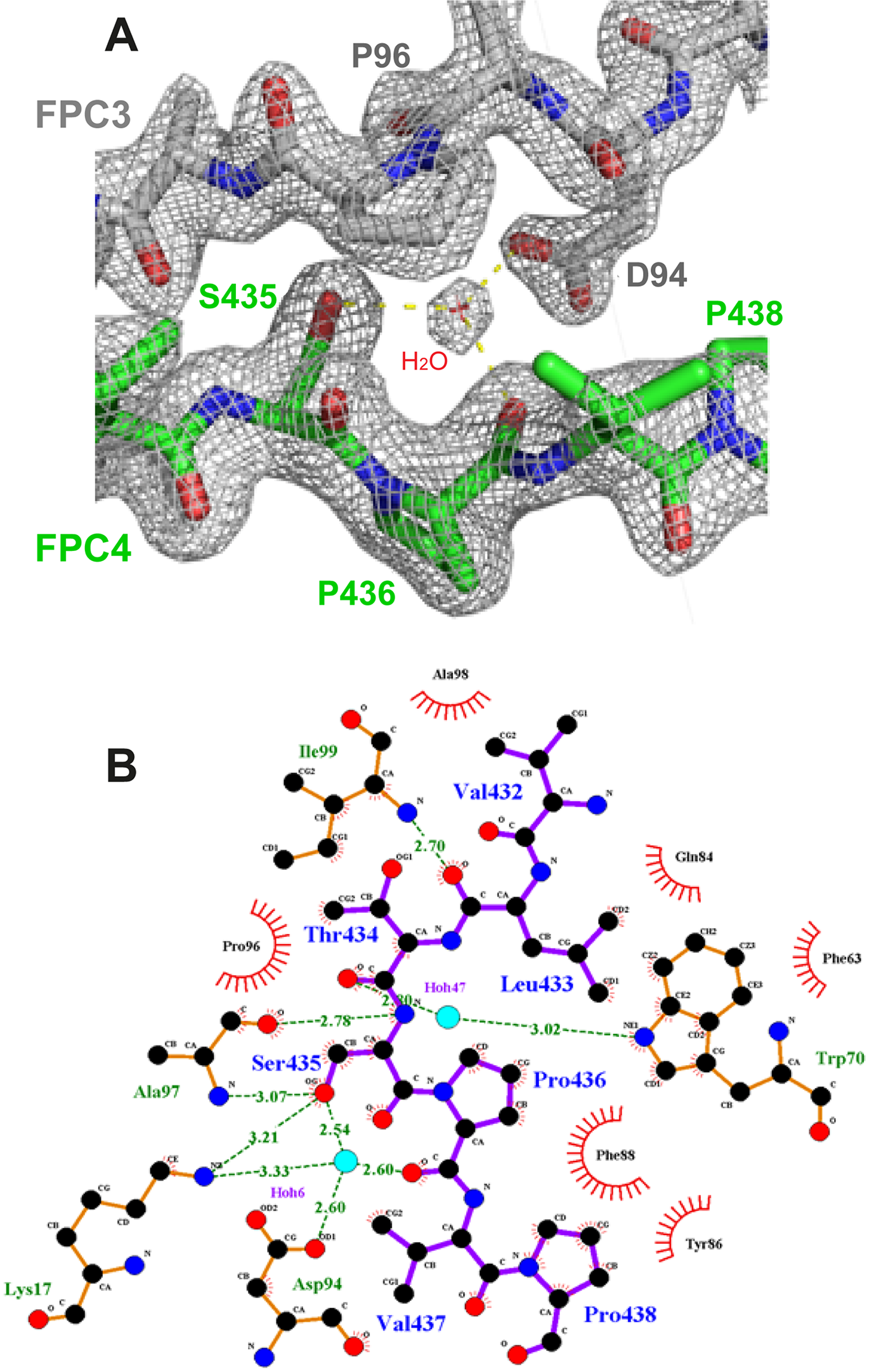

Supplement: S7 Fig — A. Zoom-in view of the central part of the interface between BILBO2 and FPC4 with the 2Fo-Fc map (grey) contoured at 1.5 σ level. An ordered water molecule form multiple hydrogen bonds with residues from both proteins. B. Details of the interaction network between BILBO2 and FPC4. The plot was generated using DIMPLOT in the LigPlot plus suite. (TIF) [file ppat.1009329.s007.tif]

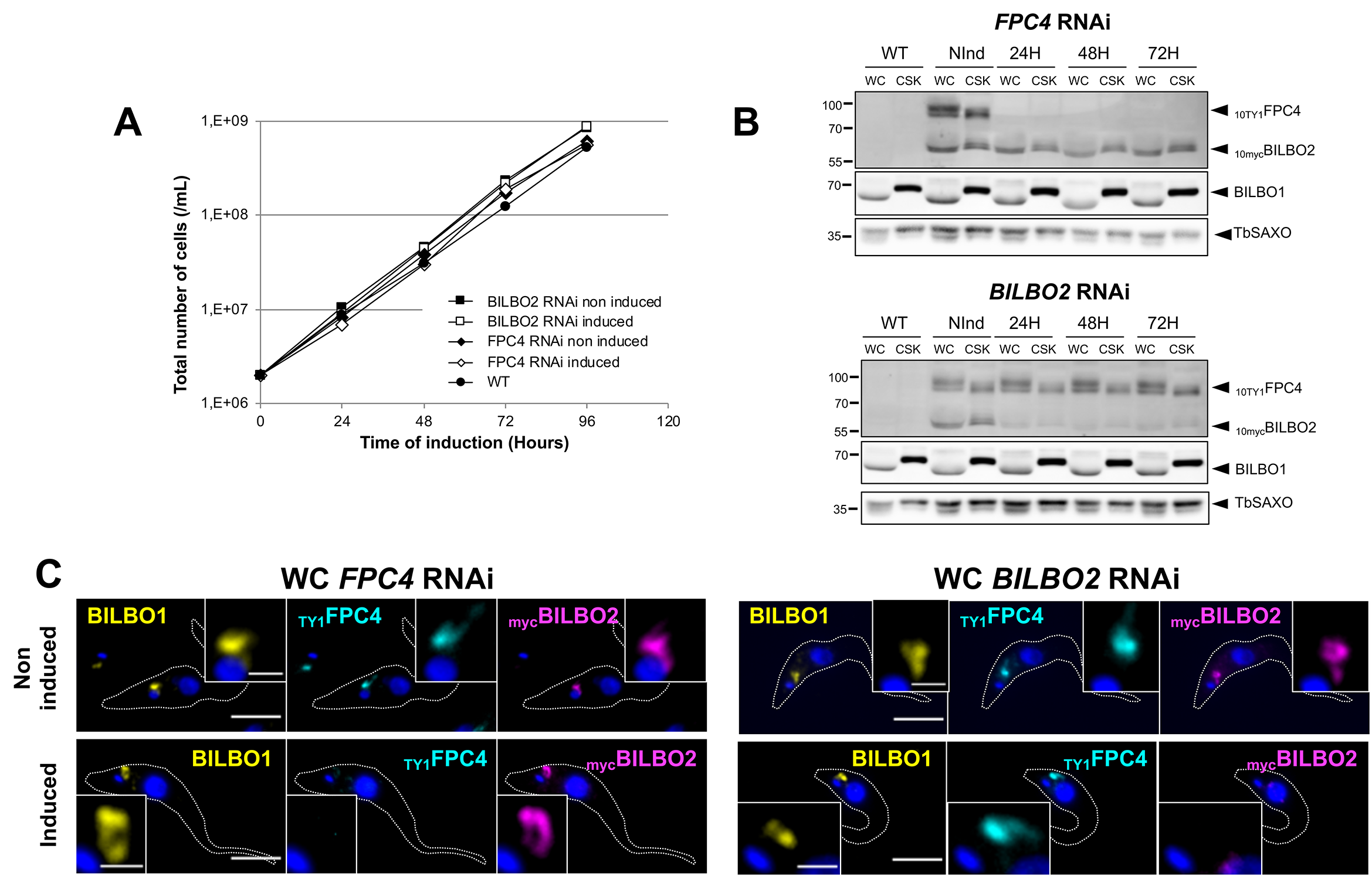

Supplement: S8 Fig — A. Growth curve of SmOxP427 cells expressing mycBILBO2 and TY1FPC4, non-induced and induced for FPC4 RNAi or for BILBO2 RNAi. B. Western blot analysis of whole cell (WC) and detergent-extracted cytoskeleton (CSK) SmOxP427 cells expressing TY1FPC4 and mycBILBO2 and induced 24-72h for FPC4 RNAi or for BILBO2 RNAi. C. Immunofluorescence labeling of BILBO1, TY1FPC4, and mycBILBO2 on whole cells 72h-induced for FPC4 RNAi or for BILBO2 RNAi. Note: no cytosolic pool was observed for FPC4 and for BILBO2 in the induced cells. Scale bars, 5 μm and 1 μm in insets. (TIF) [file ppat.1009329.s008.tif]
