# Supplementary material for: Structural and functional studies of the first tripartite protein complex at the Trypanosoma brucei flagellar pocket collar
Source: PLoS Pathog. 2021 Aug 2;17(8):e1009329. doi: 10.1371/journal.ppat.1009329 (PMC8360560; doi:10.1371/journal.ppat.1009329)
Supplement: Attachment — Submitted filename: Response to Reviewer Isch et al.pdf [file ppat.1009329.s011.pdf]

"PLOS Pathogens" plospathogens@plos.org
PLOS Pathogens: Decision on your submission PPATHOGENS-D-21-00170

Dear Dr. BONHIVERS,

Thank you very much for submitting your manuscript "Structural and functional studies of the first tripartite protein complex at the Trypanosoma brucei flagellar pocket collar" for consideration at PLOS Pathogens. As with all papers reviewed by the journal, your manuscript was reviewed by members of the editorial board and by several independent reviewers. In light of the reviews (below this email), we would like to invite the resubmission of a significantly-revised version that takes into account the reviewers' comments.

This manuscript was assessed by 3 reviewers, while all were positive about this manuscript, reviewer 2 wanted to see two additional experiments:
i) A conditional KO to investigate further whether BILBO2 is truly essential as the inability to generate a KO suggests.

ii) Further investigation of the tripartite dependencies through additional combinations of RNAi and localisation.

Given the overall reviewers positive response to this manuscript and the extensive other data within the paper we feel that i). is not necessary. However, ii). testing reciprocal FPC4 / BILBO2 localisation dependencies and hierarchies is a potentially informative set of additional experiments and given the reagents/cell lines available this is achievable in the revision timescale so you should address this.

In addition, all reviewers had a number of other points requiring textual changes that should be addressed.

We cannot make any decision about publication until we have seen the revised manuscript and your response to the reviewers' comments. Your revised manuscript is also likely to be sent to reviewers for further evaluation.

When you are ready to resubmit, please upload the following:

[1] A letter containing a detailed list of your responses to the review comments and a description of the changes you have made in the manuscript. Please note while forming your response, if your article is accepted, you may have the opportunity to make the peer review history publicly available. The record will include editor decision letters (with reviews) and your responses to reviewer comments. If eligible, we will contact you to opt in or out.

[2] Two versions of the revised manuscript: one with either highlights or tracked changes denoting where the text has been changed; the other a clean version (uploaded as the manuscript file).

Important additional instructions are given below your reviewer comments.

Dr Mélanie BONHIVERS, PhD, DR2 CNRS
Département Microbiologie Fondamentale et Pathogénicité
CNRS UMR5234, Université de Bordeaux, 146, rue Léo Saignat, 33076 Bordeaux cedex
Tél : 05 57 57 48 38 email : melanie.bonhivers@u-bordeaux.fr

Please prepare and submit your revised manuscript within 60 days. If you anticipate any delay, please let us know the expected resubmission date by replying to this email. Please note that revised manuscripts received after the 60-day due date may require evaluation and peer review similar to newly submitted manuscripts.

Thank you again for your submission. We hope that our editorial process has been constructive so far, and we welcome your feedback at any time. Please don't hesitate to contact us if you have any questions or comments.

Sincerely,

Jack Sunter Guest Editor PLOS Pathogens

David Horn Section Editor PLOS Pathogens

Kasturi Haldar Editor-in-Chief PLOS Pathogens
orcid.org/0000-0001-5065-158X

Michael Malim Editor-in-Chief PLOS Pathogens
orcid.org/0000-0002-7699-2064

****

This manuscript was assessed by 3 reviewers, while all were positive about this manuscript, reviewer 2wanted to see two additional experiments:
i) A conditional KO to investigate further whether BILBO2 is truly essential as the inability to generate aKO suggests.
ii) Further investigation of the tripartite dependencies through additional combinations of RNAi and localisation.

I feel the demonstration of BILBO2 essentiality or not will not provide additional insight and given the overall reviewers positive response to this manuscript and the extensive other data within the paper thisexperiment is not necessary. However, testing reciprocal FPC4 / BILBO2 localisation dependencies and hierarchies is a potentially informative set of additional experiments and given the reagents/cell lines available this is achievable in the revision timescale so you should address this.

In addition, all reviewers had a number of minor issues requiring textual changes that should beaddressed.

Reviewer's Responses to Questions

Part I - Summary

Please use this section to discuss strengths/weaknesses of study, novelty/significance, general executionand scholarship.

Reviewer #1: The T. brucei FPC is a vital structural component of the trypanosome cell. Despite the importance of the FPC, prior to this study, only four proteins of this structure had been identified and twocharacterised. This study identifies a further FPC protein,

Dr Mélanie BONHIVERS, PhD, DR2 CNRS
Département Microbiologie Fondamentale et Pathogénicité
CNRS UMR5234, Université de Bordeaux, 146, rue Léo Saignat, 33076 Bordeaux cedex
Tél : 05 57 57 48 38 email : melanie.bonhivers@u-bordeaux.fr

BILBO2, and convincingly demonstrates its capacity to interact with both BILBO1 and FPC4. The study also includes a crystal structure that reveals the mode of binding of BILBO2 to FPC4 and identifies the key residues involved. Together these data areof strong quality, important, novel and publishable. However, the manuscript also indicates that a molecular mechanism by which BILBO2 it is able to influence the BILBO1 filament shape has been elucidated and, in my opinion, the data does not support that conclusion.

-->We thank the reviewer for these comments and agree that our statements regarding the influence of BILBO2 on BILBO1 polymer shape could be considered confusing. We have not elucidated the molecular mechanism by which BILBO2 is able to influence BILBO1 filament shape. We had discussed the possible effects of BILBO2 on BILBO1. Now in the discussion we added the sentence "**However, we have not elucidated the molecular mechanism(s) by which BILBO2 is able to influence BILBO1 filament shape**" (line 438).

Supported claims

1) BILBO2 is a BILBO1 interacting protein, dependent on a B1BD.

The interaction is supported in two different heterologous systems; Y2H assays (Fig 1) and in U-2 OS mammalian cells (Fig 2) with the same B1BD of the BILBO2 protein necessary and sufficient for the interaction in both systems. On detergent extracted cells, the two proteins also co-localise throughout thecell cycle in T. brucei, again in a B1BD-dependent manner (Fig 3, S6, S7). This claim is therefore, well supported by the data in the manuscript.

2) BILBO2 interacts with FPC4

The interaction is supported with Y2H assays, in U-2 OS mammalian cells and through partial co- localisation of BILBO2 and FPC4 (all Fig 6). Again, domains required for interactions are consistent between methods. This interaction, and the domains responsible, are fully validated with ITC (Fig 8) and a crystal structure (Fig 7). Two residues predicted to be important for this interaction, based on the crystal structure, were additionally shown to affect protein binding by ICT and in vivo (Fig 8). This claim istherefore strongly supported by the evidence provided.

Reviewer #2: African trypanosomes are kinetoplastid parasites that cause the disease African Sleeping Sickness. The flagellar pocket is central to their pathogenicity as it is the only site of endocytosis and it sequesters the cell's surface receptors, meaning that the FP is the hub of host-parasite interaction. The Flagellar pocket collar (FPC) is essential for FP biogenesis and interfering with its function causes parasitecell death.
In this study, the authors characterise one of the hits (BILBO2) from a previous Y2H screen on BILBO1, the major structural component of the trypanosome flagellar pocket collar (FPC). They use a variety of invivo expression systems (yeast, mammalian cells and trypanosomes) to demonstrate direct interaction between specific domains of BILBO1, BLBO2 and FPC4 (termed "tripartite" by the authors here). They raise antibodies and use epitope tagging to demonstrate localisation of BILBO2 at the FPC, and also use 3-colour expansion microscopy to demonstrate almost perfect colocalisation of BILBO1 and BILBO2, with close localisation of FPC4. The authors use X-ray crystallography to solve the structure o the BILBO2- NTD/FPC4-CTD complex.
The data is high quality, the experiments innovative and well executed and, with some exceptions detailed below, the conclusions are warranted from the data. The major concern I have with the study is the absence of functional insight. No data is presented to indicate BILBO2 function, provide additional insights into FPC assembly, or that the tri-partite

Dr Mélanie BONHIVERS, PhD, DR2 CNRS
Département Microbiologie Fondamentale et Pathogénicité
CNRS UMR5234, Université de Bordeaux, 146, rue Léo Saignat, 33076 Bordeaux cedex
Tél : 05 57 57 48 38 email : melanie.bonhivers@u-bordeaux.fr

complex is important in FPC assembly. Some functionalinsights are warranted for this work to be published in PLIOS Pathogens.

Reviewer #3: Using a combination of techniques the authors identified a novel component of the flagellarpocket collar, a curious structure that is required to form the flagellar pocket, which is an membranous invagination at the base of the single flagellum of the human and animal parasite Trypanosoma brucei.
The authors very clearly demonstrate the overlapping localization with a previously described flagellar pocket collar protein (Bilbo 1) that forms a ring like structure in the FPC region. They demonstrate this insitu as well as in a human cell system where these two proteins form large filamentous complexes.

--> We indeed previously showed that BILBO1 oligomerizes into filaments using the U-2 OS cells. Here, a similar experimental setup for BILBO2 does not however support the idea that BILBO2 forms filaments (Fig. 2A,b) but only that BILBO2 docks onto the BILBO1 filaments (Fig. 2A,c). The yeast two-hybrid data suggest that BILBO2 might be able to form dimers or oligomers.

Theygo on to map the interacting domains of these two proteins and with FPC4 another protein of this structure. They furthermore show that Bilbo 2 requires Bilbo 1 for proper localization and that Bilbo 2 likely functions in shape determination of the Bilbo 1 filaments. Finally they solve the structure of the Bilbo1/2 complex and demonstrate the mechanism of interaction.

--> We solved the structure of the BILBO2-NTD / FPC4-CTD, not of the BILBO1-NTD / BILBO2-NTD complex. This is indeed a first for the FPC.

The manuscript is well written, figuresare clear and mostly with good figure legends. The data is of very high quality (I can not judge the structure data I should add). I wish I were an author on this manuscript. For the future I strongly recommend to put this amount of work in at least two PLOS Pathogens manuscripts!!

-->We appreciate the comment and thank the reviewer.

The manuscript should be accepted with minor adjustments to text and figure legends.

Part II – Major Issues: Key Experiments Required for Acceptance

Please use this section to detail the key new experiments or modifications of existing experiments thatshould be absolutely required to validate study conclusions.

Generally, there should be no more than 3 such required experiments or major modifications for a "MajorRevision" recommendation. If more than 3 experiments are necessary to validate the study conclusions, then you are encouraged to recommend "Reject".

Reviewer #1: None

Dr Mélanie BONHIVERS, PhD, DR2 CNRS
Département Microbiologie Fondamentale et Pathogénicité
CNRS UMR5234, Université de Bordeaux, 146, rue Léo Saignat, 33076 Bordeaux cedex
Tél : 05 57 57 48 38 email : melanie.bonhivers@u-bordeaux.fr

Reviewer #2: The authors propose that the lack of RNAi phenotype is due to incomplete penetrance, anduse Alsford et al RITseq data and their inability to generate a KO to suggest BILBO2 is in fact essential. However, the lack of phenotype could be because BILBO2 is not essential for FPC biogenesis (or for any other in vitro cellular function), a RITseq phenotype could be a false positive in this dataset, and the inability to generate a KO could be due to technical challenges.

--> We agree with the reviewer. The non-essentiality hypothesis is now mentioned in line 245.

To clarify this issue, the authors should make a BILBO2 KO with an inducible, ectopic addback and perform an analysis on any resulting phenotype.

--> Since we have now developed and included the non-essentiality hypothesis which is now mentioned line 245 we feel that the KO cell line is no longer required. In addition, the editor has mentioned that the KO cell line is indeed not required.

The authors propose a tri-partite protein complex at the FPC, and ablate BILBO1 to demonstrate BILBO2'sdependency upon BILBO1. However, this is a rather extreme test of dependency because the FPC is not present in BILBO1 RNAi cells, and is therefore not a very informative experiment.
To gain further insightsinto BILBO1-BILBO2-FPC4 tri-partite dependencies and hierarchies, the authors should ablate FPC4, BILBO2 and examine the affect upon BILBO2 and FPC4 localisation. It would be informative (but not absolutely necessary) to determine whether BILBO2 and FPC4 interact in the cytosol of BILBO1 ablated cells.

--> As suggested by the reviewer, we generated a SmOxP427 cell line expressing endotagged $_{TY1}$FPC4 and $_{myc}$BILBO2 and inducible for *BILBO2*, or *FPC4* RNAi and analyzed the expression level and cellular localization of $_{TY1}$FPC4 and $_{myc}$BILBO2. The results are presented in S9 Fig. and lines 393-401. Western blotting and immunofluorescence analyses showed that knockdown of FPC4 does not affect BILBO2 and *vice versa*.

Reviewer #3: (No Response)

Part III – Minor Issues: Editorial and Data Presentation Modifications

Please use this section for editorial suggestions as well as relatively minor modifications of existing datathat would enhance clarity.

Reviewer #1: The authors state on page 8 that "these data identify BILBO2 as a novel BILBO1-binding protein that plays a role in the oligomerization of BILBO1" and in the abstract "BILBO2 colocalizes with BILBO1 and can modulate the shape of the BILBO1 filament by interacting with the BILBO1 EF-hand domains". The data does not convincingly demonstrate that BILBO2 plays a role in oligomerisation of BILBO1 and therefore, although this possibility should be discussed in the discussion, it should not be presented as a finding unless the authors would like to add additional data to support this. While BILBO2 does seem to facilitate the production of short filaments in the mEFh1-expressing cells, WT

Dr Mélanie BONHIVERS, PhD, DR2 CNRS
Département Microbiologie Fondamentale et Pathogénicité
CNRS UMR5234, Université de Bordeaux, 146, rue Léo Saignat, 33076 Bordeaux cedex
Tél : 05 57 57 48 38 email : melanie.bonhivers@u-bordeaux.fr

BILBO1 is able to oligomerise without BILBO2 (Fig 2Aa), whereas BILBO2 does not oligomerise without BILBO1 (Fig2Ab / Fig S1) or localise to the FPC (Fig 4). Indeed, from the data in this paper, BILBO2 appears to require BILBO1 or FPC4 for its localisation and/or filament formation and not the other way around.

--> we agree that saying that "BILBO2 plays a role in the oligomerization of BILBO1" is misleading. Line 205 now reads as follows: "Taken together, these data identify BILBO2 as a novel BILBO1-binding protein that plays a role in the shape of the polymers formed by BILBO1 and may regulate BILBO1-mediated FPC assembly or function in the parasite.".

Other minor comments:
Figure 3 is focussed on the detergent extracted cells for co-localisation of BILBO1 and BILBO2 in these cells. For panel C the whole cells clearly have BILBO2 distributed throughout the cell as well as the FPC.This is not discussed in the text, but presumably, given the data in Fig 4, this is due to overexpression ofthe ectopically-expressed BILBO2. That should be stated in the text (ideally along with an accompanying western blot to demonstrate extent of overexpression).

--> We used western blotting to assess the expression level of WT and of the induced tagged $BILBO2_{HA}$ (anti-BILBO2 polyclonal antibody and anti-HA tag antibody). The labeling showed that, indeed, $BILBO2_{HA}$ and the other domains (excepting $B1BD_{HA}$) are overexpressed compared to the endogenous WT BILBO2 generating a cytosolic pool that is extracted during detergent treatment. The corresponding results are now presented in the text (lines 219-223) and in S3 Fig.B.

Figure 3 legend has panel C and D descriptions the wrong way round.
--> corrected.

Figure 4B: 48 hour cell should include inset panel (as for the NI and 24 hour cells) to support statement on page 10 that "…after 48h of induction BILBO2 was neither detected at the old flagellum nor at the newflagellum".
-->inset panels were added.

No scale bar on inset panels of Figures 4B or 5B.
-->scale bars were present but too thin, they are now thicker.

Resolution of figures in reviewers PDF are lower than publication quality and hard to read small text,especially Fig 6. --> Some text has been enlarged in 1A.

Material spelt incorrectly on page 18.
--> corrected.

Line numbers should have been included for the review process.
--> we apologize for the inconvenience; in this new version, we added line numbers.

Reviewer #2: Figure 3 quantitation suggests that BILOBO2 protein intensity is half that of BILBO1 in latecell cycle stages, but this is not apparent on the example micrographs. Presumably this is an issue of contrast settings - it would be helpful for the reader to have the same contrast settings on early and latecells so the reduction in signal intensity can be observed.

Dr Mélanie BONHIVERS, PhD, DR2 CNRS
Département Microbiologie Fondamentale et Pathogénicité
CNRS UMR5234, Université de Bordeaux, 146, rue Léo Saignat, 33076 Bordeaux cedex
Tél : 05 57 57 48 38 email : melanie.bonhivers@u-bordeaux.fr

--> To exemplify more clearly the decrease in BILBO2 level at this cell cycle stage in Figure 3B, we have chosen another 2K1N image that is more representative.

More details should be provided (in the text of Figure legend) on what is meant by BILBO1 and 2 fluorescence intensity at the old and new FPC. i.e. is this sum intensity per collar, or average intensity perpixel?
-->This is now provided in the figure 3 legend.

Figure 3D and 3C are mixed up in the Figure legend.
-->corrected.

In figure 3, the data and text on over-expression of BILBO2-T2 in trypanosomes is confusing and contradictory. The text states that BILBO2-T2 localises to the FPC in whole cells but is mostly extracted by detergent, concluding that this mutant has FPC localisation but is not fully functional. However, I am unable to see this mutant localise to the FPC on whole cells. In contrast, this mutant DOES localise clearlyand brightly to the FPC in cytoskeletons, but only in the enlarged inset, and it is not evident at all in the main panel. This discrepancy in the text and data need to be resolved.

-->We agree that this figure can be misleading. We now used western blotting to assess the expression level of WT and the induced tagged BILBO2 (anti-BILBO2 polyclonal antibody and anti-HA tag antibody) (S3 Fig.B, C). The labeling showed that $BILBO2_{HA}$ and the other domains (except $B1BD_{HA}$) are overexpressed compared to the endogenous BILBO2 generating a cytosolic pool that is extracted during detergent treatment. The corresponding results are now presented in the text (lines 219-223) and in the S3 figure. In the IF experiments, T1 and T2 are removed during the extraction, but a weak and consistent labelling of T2 was observed at the FPC. This labeling is difficult to evidence on a figure. The contrast in the inset has been increased in both T1 and T2; this is now indicated in the figure legend. We wish to keep this data because we don't want to miss a potential interesting information on the role of the NTD-B1BD linker domain present in T2.

Figure 4C – typo (Tubuline should be Tubulin)
-->corrected

Figure 5A legend states that asterisks indicate identical residues – this is not true in several cases whenexamining the alignment.
--> We apologies for the residues' shift that was presented in Fig 5A. This is now corrected and identical residues align.

Figure 5 legend typo – columns should be colons
-->corrected

Figure 5 B legend – amend to state these are cytoskeletons, rather than whole cells
-->corrected

Figure 5B and associated text: The authors convincingly demonstrate that the BILBO1/2 NTD chimeras localise to the FPC in cytoskeletons, although their experiment would have been more compelling if they had used anti-Ty labelling of Ty::BILBO1 to prevent anti-BILBO1 cross-reactivity with the chimera in theircolocalisation fluorescence microscopy. The authors previously show (Figure 3C) that B1BD is necessary and sufficient for FPC localisation, and the localisation of BILBO1 to the FPC has not been shown to require its NTD. Therefore, there is nothing to suggest that the localisation of these mutant proteins to

Dr Mélanie BONHIVERS, PhD, DR2 CNRS
Département Microbiologie Fondamentale et Pathogénicité
CNRS UMR5234, Université de Bordeaux, 146, rue Léo Saignat, 33076 Bordeaux cedex
Tél : 05 57 57 48 38 email : melanie.bonhivers@u-bordeaux.fr

the FPC is due to the presence of the chimeric BILBO1/2 NTDs. Moreover, the authors state their data

supports the hypothesis that the BILBO1/2 NTDs share a similar function. However, they only presentlocalisation data, not functional data, so no functional inferences can be drawn. For functional insights,the authors must demonstrate that the chimeras can perform the function of the wildtype protein (e.g.rescue of an RNAi phenotype).

-->Following the suggestion of the reviewer, we have generated a cell line inducible for *BILBO1* RNAi and for ectopic expression of recoded version of BILBO1$_{HA}$ and $_{Ch}$BILBO2-BILBO1$_{HA}$ (that are not targeted by *BILBO1* RNAi). Cells were induced with tetracycline at 2ug/mL (minimum concentration of tetracycline to observe the RNAi phenotypes in the parental *BILBO1* RNAi cell line). The cellular growth and the expression of wild-type BILBO1 and of recoded proteins were assessed. Recoded BILBO1$_{HA}$ is able to fully rescue the *BILBO1* RNAi phenotypes. However, the recoded chimeric BILBO2-BILBO1$_{HA}$ protein was not able to rescue the *BILBO1* RNAi. So the NTD are not exchangeable *in vivo*. We have integrated these new data in Fig. 5C and lines 295-301 and 473-474.

Figure 6A: regarding the second Y2H in the series – could the authors confirm whether this was intendedto indicate be a 3-way Y2H, between BILBO2-NTD, BILBO2-B1BD, and FPC4-B1BD?
-->A linker was missing between BILBO2 NTD and B1BD suggesting a 3-way Y2H. It is a 2-way test and the linker is now present on the figure.

Reviewer #3: Minor changes in the figures/figure legends:
Figure 1 B: I am not sure I understand the fourth row where B1BD is tested as prey and bait? Also the
-Control and +Control are not well explained in the figure legends. Also please stay consistent with thenaming scheme Bilblo1-BD and B1BD is the same I assume no need to have different names then.?

-->We agree that Figure 1B was unclear. We have modified the fourth row to better explain that BILBO2 was cloned in both prey and bait vectors. The resulting interaction assay suggests that BILBO2 could form a homodimer. Figure 1B legend was modified for more clarity about the internal controls. B1BD is now uniform in the figures.

Figure 3:

- C and D are reversed in comparison to their description in the figure legend?
  --> Indeed, the legend was inversed and we corrected it.

- In the figure legend part C, please mention the number of cells this has been quantified with?
  -->We added the number of cells (n=200).

- Figure 6:
- In figure 6 C, please double-check the scale bars. It is decribed to be 5um, but with expansion, the cellsshould be much bigger than that I expect
-

-->We apologize to the reviewers. In the initially presented scale bars, we took into account the expansion factor (x4.3). This was misleading. We now present the figure with the scale bar calculated from the camera's pixel size (10um in the main image and 5um in the enlarged inset).

Dr Mélanie BONHIVERS, PhD, DR2 CNRS
Département Microbiologie Fondamentale et Pathogénicité
CNRS UMR5234, Université de Bordeaux, 146, rue Léo Saignat, 33076 Bordeaux cedex
Tél : 05 57 57 48 38 email : melanie.bonhivers@u-bordeaux.fr

Minor changes in the text:

- Page 2 text passage "…demonstrate that BILBO1 and BILBO2 share a homologous NTD domain and thatboth domains…": delete the word domain. That word is already part of the abbrevation NTD. ?

-->This has been corrected.

- Page 9/10 the passage about the BILBO2 knockdown. The authors explain that RNAi of BILBO2 had no effect, and they give the remaining trace amounts of BILBO2 as a possible explanation for this behaviour.In their data, they give some evidence that to form a flagellar pocket, the parasite indeed doesn't need 100% of the BILBO2 a G1 cell has (Figure 3D). They show that a signal of 50% per flagellar pocket collar in 1K1N-2FPC, as observed in IFA, is enough for the pocket to build. This supports the idea that an incomplete knockdown would not be enough to efficiently disrupt flagellar pocket collar formation. Thus, the assumption of the authors that the incomplete depletion of BILBO2 is the reason for the missing phenotypical effect, is true. But they should make use of their own data to support their conclusion. -->We thank the reviewer for this comment and we have integrated it in the text (lines 418-421).

- Page 12 teyt passage "…probably due to primary and secondary antibodies steric hindrance, with turnedto…": Typo, with = we

--> corrected.

Please do not refer to other publications for the methods. Describe the methods in the manuscript andadd references where appropriate (in particular for the Yeast two-hybrid assay).

-->an online protocol has been submitted to protocol.io which DOI is now mentioned line 693.

Please Adjust the reference for Amodeo et al. 2020 to the now published version: Journal of Cell Science2021 : jcs.254300 doi: 10.1242/jcs.254300 Published 15 February 2021

--> corrected

PLOS authors have the option to publish the peer review history of their article (what does this mean?). Ifpublished, this will include your full peer review and any attached files.

If you choose "no", your identity will remain anonymous but your review may still be made public.

Do you want your identity to be public for this peer review? For information about this choice,including consent withdrawal, please see our Privacy Policy.

Reviewer #1: No

Reviewer #2: No

Reviewer #3: No

Figure Files:

Dr Mélanie BONHIVERS, PhD, DR2 CNRS
Département Microbiologie Fondamentale et Pathogénicité
CNRS UMR5234, Université de Bordeaux, 146, rue Léo Saignat, 33076 Bordeaux cedex
Tél : 05 57 57 48 38 email : melanie.bonhivers@u-bordeaux.fr

While revising your submission, please upload your figure files to the Preflight Analysis and Conversion Engine (PACE) digital diagnostic tool, https://pacev2.apexcovantage.com. PACE helps ensure that figuresmeet PLOS requirements. To use PACE, you must first register as a user. Then, login and navigate to the UPLOAD tab, where you will find detailed instructions on how to use the tool. If you encounter any issuesor have any questions when using PACE, please email us at figures@plos.org.

Data Requirements:

Please note that, as a condition of publication, PLOS' data policy requires that you make available all dataused to draw the conclusions outlined in your manuscript. Data must be deposited in an appropriate repository, included within the body of the manuscript, or uploaded as supporting information. This includes all numerical values that were used to generate graphs, histograms etc.. For an example see here on PLOS Biology: http://www.plosbiology.org/article/info%3Adoi%2F10.1371%2Fjournal.pbio.1001908#s5.
--> Raw data for the quantifications in figures 3D and 4D are now provided in S10 Data.

Reproducibility:
To enhance the reproducibility of your results, PLOS recommends that you deposit laboratory protocols inprotocols.io, where a protocol can be assigned its own identifier (DOI) such that it can be cited independently in the future. For instructions see http://journals.plos.org/plospathogens/s/submission- guidelines#loc-materials-and-methods
--> The yeast two-hybrid assay protocol is now detailed in an online protocol. The DOI is mentioned in line 693.

Dr Mélanie BONHIVERS, PhD, DR2 CNRS
Département Microbiologie Fondamentale et Pathogénicité
CNRS UMR5234, Université de Bordeaux, 146, rue Léo Saignat, 33076 Bordeaux cedex
Tél : 05 57 57 48 38 email : melanie.bonhivers@u-bordeaux.fr
