# Supplementary material for: Structural and functional studies of the first tripartite protein complex at the Trypanosoma brucei flagellar pocket collar
Source: PLoS Pathog. 2021 Aug 2;17(8):e1009329. doi: 10.1371/journal.ppat.1009329 (PMC8360560; doi:10.1371/journal.ppat.1009329)
Supplement: Attachment — Submitted filename: Response to Reviewer .docx [file ppat.1009329.s012.docx]

Dear Editor,

Thank you for giving us the opportunity to improve our manuscript number PPATHOGENS-D-21- 00170. We took into account the most recent comments of reviewer 1. We have modified the abstract and the sentence line 463 :

Thank you very much for submitting your revised manuscript "Structural and functional studies of the first tripartite protein complex at the Trypanosoma brucei flagellar pocket collar" for consideration at PLOS Pathogens. We are likely to accept this manuscript for publication, but ask that you address the points below and modify the manuscript accordingly.

 The abstract needs to be reworked to reflect the comments by reviewer 1 on the effect of BILBO2 on BILBO1 polymers. Currently, it states "... and can modulate the shape of the BILBO1 filament by interacting with the BILBO1 EF-hand domains." which appears to be too strong a conclusion from the evidence presented.

- We have removed the sentence “We show that BILBO2 colocalizes with BILBO1 and can modulate the shape of the BILBO1 filament by interacting with the BILBO1 EF-hand domains.” And reworked the previous sentence which now reads as “Here, we report by yeast two-hybrid, bioinformatics, functional and structural studies the characterization of a new FPC component and BILBO1 partner protein, BILBO2 (Tb927.6.3240). “

Plus on line 463 it should be unknown.

- “Poorly known” was modified to unknown.

We hope that PLoS Pathogens will consider the revised version of the manuscript suitable for publication.

Sincerely,

Dr. Mélanie Bonhivers

Dear Dr. BONHIVERS,
